# Paediatric Allergic Asthma: Risk Factors, Diagnosis, Control, and Treatment

**DOI:** 10.3390/children12060713

**Published:** 2025-05-30

**Authors:** Cristiana Indolfi, Angela Klain, Michele Miraglia del Giudice, Maria Angela Tosca

**Affiliations:** 1 Department of Woman, Child and General and Specialized Surgery, University of Campania ‘Luigi Vanvitelli’, 80138 Naples, Italy; cristiana.indolfi@policliniconapoli.it (C.I.); michele.miragliadelgiudice@unicampania.it (M.M.d.G.); 2 Allergy Center, IRCCS Istituto Giannina Gaslini, 16147 Genoa, Italy; mariangelatosca@gaslini.org

This Special Issue, titled “Pediatric Allergic Asthma: Risk Factors, Diagnosis, Control, and Treatment”, presents a comprehensive collection of articles that explore the complexities of asthma, which continues to impact millions of children around the world. Allergic asthma, a chronic inflammatory disorder of the airways, is particularly prevalent in children, and its global prevalence has been steadily increasing. The aim of this Special Issue is to examine various aspects of pediatric allergic asthma, including risk factors, environmental triggers, diagnosis, management, and treatment strategies. Using a multidisciplinary approach, the featured articles offer valuable insights into the disease, providing both clinical perspectives and research-driven recommendations designed to improve outcomes for children living with asthma.

## 1. Introduction: The Growing Burden of Pediatric Allergic Asthma

Asthma is one of the most common chronic diseases in children. It is characterized by airway inflammation, leading to recurrent episodes of wheezing, shortness of breath, and cough [1]. The global prevalence of asthma is continuing to rise. As reported by the World Health Organization (WHO), asthma affected an estimated 262 million people in 2019 and was responsible for 455,000 deaths [2]. According to recent studies, approximately 14% of children are affected by asthma, with allergic asthma being the dominant form in pediatric populations [3,4,5]. Triggers include environmental factors such as allergens (house dust mites (HDMs), pollen, pet dander, and mold), viral infections, smoke exposure, and air pollutants. Asthma is recognized as a complex condition, with variability in its severity and response to treatment. It often coexists with a range of comorbidities, including both atopic (allergic rhinitis, atopic dermatitis, and food allergies) and non-atopic symptoms (anxiety, depression, gastro-esophageal reflux disease, and obesity) [6,7]. The heterogeneous nature of asthma in children is evident, with different phenotypes, including allergic and non-allergic asthma, now referred to as T2-high and T2-low, respectively, showing distinct patterns in terms of triggers, severity, and responsiveness to treatment [8]. These phenotypes include various endotypes, referring to specific biological subtypes of the disease. This identification enables clinicians to tailor therapies, such as biologics, to specific inflammatory pathways, improving disease control and patient outcomes. Therefore, it is essential that the medical community continues to refine diagnostic criteria and treatment strategies to provide the best care for children with asthma.

## 2. Environmental Risk Factors and Triggers in Pediatric Asthma

One of the most well-established contributors to allergic asthma in children is exposure to environmental allergens. HDMs, a common indoor allergen, are particularly significant in the development and exacerbation of asthma, especially in genetically predisposed children. The review by Klain (contribution 1) et al. holds significant importance as this subject matter is of considerable clinical relevance. HDMs are recognized as the primary respiratory allergens globally, particularly affecting children with asthma. Due to their ubiquitous presence in indoor environments, especially in bedding and upholstered furniture, HDMs constitute a major environmental risk factor for both the onset and exacerbation of asthma. By offering practical preventive measures, this review provides essential insights into how reducing HDM exposure can effectively manage asthma symptoms and improve the overall quality of life for affected pediatric populations. Moreover, the review highlights the therapeutic role of allergen-specific immunotherapy (AIT) in accordance with the current recommendations of the Global Initiative for Asthma (GINA). It specifically suggests the consideration of sublingual immunotherapy (SLIT) for symptomatic HDM-sensitive adults or adolescents with partly controlled asthma despite the use of inhaled corticosteroids (ICs), provided that their Forced Expiratory Volume in 1 s (FEV1) is greater than 70% of the predicted value [9].

Furthermore, the contribution by Wypych-Ślusarska et al. (contribution 2) investigates the complex association between pet ownership and respiratory health in children, emphasizing the risk of reverse causality in cross-sectional studies. The study found a lower prevalence of asthma and wheezing among children currently or previously exposed to pets but cautioned that such findings cannot be interpreted as causative due to the study design’s limitations. The analysis suggests that families of asthmatic children may be more likely to avoid pet ownership, leading to the potential misinterpretation of protective effects. This work underscores the need for longitudinal research to clarify the role of animal allergens in pediatric asthma.

In addition to indoor allergens, environmental factors such as meteorological conditions also play a major role in asthma exacerbations. The study by Boura et al. (contribution 3) explored the impact of climate variables, including temperature fluctuations, wind direction, and humidity, on pediatric asthma. The research found that changes in climate and air quality have a significant impact on asthma symptoms, with children being particularly susceptible to these variations. This emphasizes the need to account for weather patterns when assessing asthma triggers in children and calls for public health policies that address environmental pollution and allergen exposure to reduce asthma exacerbations. Furthermore, exposure to outdoor pollutants, particularly traffic-related air pollution, has been shown to increase both the risk of developing asthma and the severity of existing symptoms. Air pollution and climate change are, therefore, critical factors contributing to the rising incidence of pediatric asthma. Prolonged exposure to pollutants such as nitrogen dioxide (NO₂) and fine particulate matter (PM2.5) can impair lung development and increase the risk of respiratory diseases in children. Additionally, climate change, with rising temperatures and more frequent extreme weather events, alters the distribution and concentration of allergens, exacerbating and perpetuating asthma symptoms [10,11].

## 3. Diagnosis, Management, and Monitoring of Asthma in Children

Diagnosing asthma in children can be particularly challenging, especially in younger patients who may not have the ability to clearly communicate their symptoms or undergo pulmonary function tests. This is further complicated by the fact that asthma symptoms can overlap with those of other respiratory conditions, making accurate diagnosis critical. Poorly controlled asthma, along with a lack of good basic asthma care, can exacerbate these challenges. Alarmingly, in a British report, fewer than 25% of children with asthma noted having a personalized asthma action plan [12].

The clinical diagnosis of asthma is typically based on detailed medical histories, physical examinations, and the presence of characteristic symptoms such as wheezing, coughing, and shortness of breath. In cases where asthma is suspected, lung function tests can be crucial in confirming the diagnosis [13]. Spirometry is an important diagnostic test that assesses the level of airflow obstruction and reversibility after bronchodilator administration. Spirometry is generally feasible in children aged 6 years and older. For children under the age of 5, diagnosis is often made based on medical history, a trial of asthma medication, and response to bronchodilators.

Another useful test in the diagnosing and follow-up of asthma is the Fractional Exhaled Nitric Oxide (FeNO) test, which measures the level of nitric oxide in exhaled breath. High levels of FeNO, such as >20 ppb, are typically associated with eosinophilic airway inflammation, which is common in asthma [14]. This test can be particularly useful in confirming asthma in children where symptoms are suggestive, but other tests are not conclusive, such as monitoring an individual’s adherence to inhaled corticosteroid (IC) therapy. In addition to FeNO and spirometry, peak flow monitoring can also be used to assess asthma in children [15].

When evaluating respiratory function in pediatric asthma, it is crucial to assess not only physiological parameters but also asthma control, quality of life, and psychological well-being. Tools such as the Asthma Control Questionnaire (ACQ) and the Asthma Control Test (ACT) are widely used to monitor the frequency of symptoms, medication use, and activity limitations. The ACQ, in particular, has demonstrated strong predictive validity for future exacerbations and is considered more reliable than physician-based assessments or impairment-focused tools. For a more holistic evaluation, these control measures should be supplemented with instruments like the Pediatric Asthma Quality of Life Questionnaire (PAQLQ) and the Pediatric Asthma Caregiver’s Quality of Life Questionnaire (PACQLQ). These tools provide insights into the broader impact of asthma on children’s daily functioning and emotional state, as well as caregiver burden [16].

While the physical symptoms of asthma are well-documented, the psychological impact of the disease on children and their families is often overlooked. Asthma can lead to significant emotional distress, particularly for children who experience frequent exacerbation or who are restricted from participating in physical activities due to their condition. Anxiety about potential asthma attacks, school absences, and the fear of hospitalization can negatively impact a child’s quality of life. Moreover, the emotional burden extends to parents, who frequently worry about their child’s health and long-term outcomes. Studies, including that by Rogulj et al. (contribution 4), have shown that children with asthma and their parents often experience heightened levels of anxiety, especially regarding fears related to asthma attacks during physical activities, school absences, and the fear of an asthma attack occurring without warning. Specifically, children with moderate or severe asthma reported higher levels of anxiety due to restrictions brought about by their asthma symptoms, with the greatest anxiety occurring during physical activity. Parents also reported significant fears, such as the potential for an asthma attack without warning, drug side effects, and their child missing school. These emotional concerns can further exacerbate the burden of asthma on both the child and the family, highlighting the importance of addressing both the physical and psychological aspects of asthma management.

## 4. Treatment of Pediatric Asthma

The treatment of asthma in children requires a highly individualized approach, considering the severity of the disease, its triggers, and the response to previous treatments. According to the 2024 GINA recommendations, the management of asthma should involve a stepwise approach that incorporates both pharmacologic and non-pharmacologic interventions [9]. The first-line treatment for asthma control in children is ICS, which is effective in reducing inflammation and preventing symptoms. For children who continue to experience asthma symptoms despite ICS treatment, long-acting beta-agonists (LABAs) may be added as an adjunct therapy to improve symptom control and reduce the frequency of exacerbations. Leukotriene modifiers, such as montelukast, are another option for children with mild-to-moderate persistent asthma. However, a subset of patients fails to achieve adequate control despite optimal therapy. These children often experience frequent exacerbations, require systemic corticosteroids, and suffer from impaired quality of life due to persistent symptoms, school absences, and emotional distress. Repeated systemic corticosteroid use carries significant risks, including growth suppression, adrenal suppression, and increased infection susceptibility.

The recognition that allergic asthma is driven by T2-high inflammation has paved the way for biologic therapies, including monoclonal antibodies that interrupt key immunologic mechanisms. Omalizumab, the first biologic approved for asthma, targets circulating immunoglobulin E (IgE), preventing it from binding to receptors on mast cells and basophils. This action dampens the allergic cascade and reduces exacerbation risk [17]. Clinical trials have shown that omalizumab significantly reduces ICS requirements and school absences while improving lung function in children aged ≥6 years with moderate-to-severe allergic asthma. Patient selection for omalizumab is based on serum IgE levels (30–1500 IU/mL) and evidence of perennial allergen sensitization. Its safety profile is favorable, with most adverse effects being mild injection site reactions or upper respiratory symptoms [18].

Newer biologics targeting interleukin (IL)-5 and IL-5 receptor alpha (IL-5Rα), such as mepolizumab and benralizumab, offer additional options for allergic asthma with eosinophilic inflammation. These therapies reduce eosinophil survival and activation, effectively lowering exacerbation rates and corticosteroid needs. While mepolizumab has been approved for children aged ≥6 years, benralizumab has recently gained approval for the same age group in select regions. Both agents are administered subcutaneously, with extended dosing intervals improving adherence in pediatric care [19].

Dupilumab, a monoclonal antibody that blocks the IL-4 receptor alpha (IL-4Rα), simultaneously inhibits IL-4 and IL-13 signaling, which are two central drivers of allergic asthma. It has demonstrated robust efficacy in children with moderate-to-severe asthma, particularly those with elevated eosinophils and/or FeNO levels. Its use is also beneficial in patients with comorbid atopic dermatitis or chronic rhinosinusitis with nasal polyps (CRSwNP), providing a comprehensive T2 anti-inflammatory strategy [20]. Dupilumab is administered via subcutaneous injection, with dosing based on age and body weight, typically every two or four weeks.

Tezepelumab, a newer biologic targeting thymic stromal lymphopoietin (TSLP), represents an important advancement in the treatment of severe asthma, including in pediatric populations. Unlike other biologics that target downstream T2 cytokines, tezepelumab acts upstream in the inflammatory cascade and has demonstrated efficacy across a broader spectrum of asthma phenotypes, including T2-low disease. Recent trials show that tezepelumab significantly reduces asthma exacerbations and improves lung function in adolescents, offering a promising option for patients who are not eligible for or responsive to other biologics [21]. Tezepelumab is administered via subcutaneous injections at a dosage of 210 mg once every 4 weeks.

Despite these advances, the implementation of biologics in pediatric asthma must be guided by careful patient selection. Biomarkers such as blood eosinophil count, FeNO, and serum IgE levels help stratify children who are most likely to benefit. Practical factors, including age, comorbidities, and treatment burden, also play a role in therapy selection [22]. Monitoring the response at 4–6 months is essential, with discontinuation considered in well-controlled patients after extended use, though long-term safety data are still evolving. In the article by Miculinic et al. (contribution 5), the authors discuss recent advances in asthma diagnosis, focusing on new diagnostic tools and techniques that can improve early detection. According to the authors, the use of biomarkers, such as FeNO, and advanced imaging methods have shown promise in diagnosing asthma with greater precision. These technologies can help identify asthma in its early stages, allowing for timely intervention and better management of the disease. Genetic testing may also offer insights into how asthma will progress, providing further opportunities for personalized treatment plans.

In summary, the treatment of pediatric allergic asthma has entered a new era defined by targeted, individualized therapy. Biologic agents offer a meaningful clinical improvement for children who remain symptomatic despite conventional therapies, and their expanding indications promise further gains in pediatric asthma care. Ongoing research will help refine treatment algorithms, improve biomarker-guided decision-making, and ensure safe, effective, and equitable access to these life-changing therapies.

## 5. Conclusions: Moving Forward in Pediatric Allergic Asthma Care

This Special Issue highlights the importance of a multidisciplinary approach to pediatric allergic asthma, one that incorporates insights from environmental science, genetics, psychology, and clinical research. As we look to the future, continued collaboration and innovation will be necessary to improve the lives of children living with asthma and reduce the global burden of this chronic disease.
